# Imaging of Intratumoral Inflammation during Oncolytic Virotherapy of Tumors by ^19^F-Magnetic Resonance Imaging (MRI)

**DOI:** 10.1371/journal.pone.0056317

**Published:** 2013-02-18

**Authors:** Stephanie Weibel, Thomas Christian Basse-Luesebrink, Michael Hess, Elisabeth Hofmann, Carolin Seubert, Johanna Langbein-Laugwitz, Ivaylo Gentschev, Volker Jörg Friedrich Sturm, Yuxiang Ye, Thomas Kampf, Peter Michael Jakob, Aladar A. Szalay

**Affiliations:** 1 Department of Biochemistry, Biocenter, University of Wuerzburg, Wuerzburg, Germany; 2 Department of Experimental Physics 5, University of Wuerzburg, Wuerzburg, Germany; 3 Research Center for Magnetic Resonance Bavaria e.V., Wuerzburg, Germany; 4 Rudolf Virchow Center, Research Center for Experimental Biomedicine, and; 5 Institute for Molecular Infection Biology, University of Wuerzburg, Wuerzburg, Germany; 6 Department of Radiation Medicine and Applied Sciences, University of California San Diego Health System, La Jolla, California, United States of America; 7 Genelux Corporation, San Diego Science Center, San Diego, California, United States of America; Mayo Clinic, United States of America

## Abstract

**Background:**

Oncolytic virotherapy of tumors is an up-coming, promising therapeutic modality of cancer therapy. Unfortunately, non-invasive techniques to evaluate the inflammatory host response to treatment are rare. Here, we evaluate ^19^F magnetic resonance imaging (MRI) which enables the non-invasive visualization of inflammatory processes in pathological conditions by the use of perfluorocarbon nanoemulsions (PFC) for monitoring of oncolytic virotherapy.

**Methodology/Principal Findings:**

The Vaccinia virus strain GLV-1h68 was used as an oncolytic agent for the treatment of different tumor models. Systemic application of PFC emulsions followed by ^1^H/^19^F MRI of mock-infected and GLV-1h68-infected tumor-bearing mice revealed a significant accumulation of the ^19^F signal in the tumor rim of virus-treated mice. Histological examination of tumors confirmed a similar spatial distribution of the ^19^F signal hot spots and CD68^+^-macrophages. Thereby, the CD68^+^-macrophages encapsulate the GFP-positive viral infection foci. In multiple tumor models, we specifically visualized early inflammatory cell recruitment in Vaccinia virus colonized tumors. Furthermore, we documented that the ^19^F signal correlated with the extent of viral spreading within tumors.

**Conclusions/Significance:**

These results suggest ^19^F MRI as a non-invasive methodology to document the tumor-associated host immune response as well as the extent of intratumoral viral replication. Thus, ^19^F MRI represents a new platform to non-invasively investigate the role of the host immune response for therapeutic outcome of oncolytic virotherapy and individual patient response.

## Introduction

Oncolytic virotherapy of tumors is based on the lytic destruction of solid tumors mediated by infection of the malignant tissue by tumor-specific viruses [1]–[4]. Today, a multitude of different virus strains with oncolytic potential are described in the literature and promising pre-clinical data as well as clinical trial reports from oncolytic virotherapy are available [5]–[7]. In addition to the oncolytic tissue destruction massive inflammation within the tumor microenvironment occurs, which is primarily directed against the virus [8]–[10]. However, virus-mediated cell lysis also leads to the release of tumor-associated antigens, which may finally stimulate an anti-tumoral immune response [11], [12]. So far, it is well known that oncolytic virotherapy is accompanied by a host immune response, however, the individual time course, the polarization of the immune response, and the effect on the therapeutic efficacy remain to be elucidated.

In practice, a longitudinal, non-invasive quantification of the intratumoral inflammation during oncolytic virotherapy may provide substantial benefits to therapeutic monitoring, tumor diagnostics and indirect virus imaging as well as to the optimization of new therapeutic virus strains. Recently, ^19^F/^1^H MRI was introduced as a novel and promising non-invasive imaging modality of different inflammatory conditions in rodent models of cardiac ischemia [13], allograft rejection [14], LPS-induced pulmonary inflammation [15], inflammatory bowel disease [16], neuro-inflammation [17] and bacterial abscess formation [18]. In practice a single intravenous injection of an emulsified perfluorocarbon (PFC) predominantly lead to the phagocytic uptake by the monocyte-macrophage system followed by their detection via ^19^F MRI [19]. As a consequence of the progressive infiltration of PFC-labeled immune cells into inflamed tissues, the foci of inflammation can be localized as hotspots and morphologically correlated to the anatomical or patho-physiological context with the help of ^1^H images [19], [20].

Although recent advances in the visualization of inflammatory processes using different imaging modalities were reported [21]–[23], ^19^F MRI has several advantages especially compared to contrast agent based ^1^H MRI. First, no detectable fluorine signal is present in tissues enabling background-free ^19^F MRI of the exogenous ^19^F marker [20] and the directly acquired ^19^F signal facilitates the quantification of the marker amount [24]. Second, the ^1^H signal is not altered compared to other contrast agents making the assessment of other quantitative ^1^H parameters feasible [18]. Therefore, ^19^F MRI is a promising modality for imaging of inflammation combined with high anatomical resolution (^1^H) in the context of oncolytic virotherapy.

The abundance of inflammatory cells is a hallmark of cancer, and their importance in cancer development and progression have been demonstrated in numerous studies [25], [26]. Of particular importance are macrophages due to their high phenotypic heterogeneity reaching from cytotoxic M1-polarized macrophages, which can theoretically harm tumor tissues to M2-polarized macrophages (tumor-associated macrophages, TAMs), which promote tumorigenesis [27]. The destruction of TAMs or the reprogramming of tumor-promoting M2 to cytotoxic M1 macrophages represents a current immunotherapeutic strategy against cancer [28]. Since colonization of solid tumors with systemically injected oncolytic viruses induces macrophage-recruitment this treatment may interfere with the polarization of macrophages by confronting the host with a variety of pathogen-associated molecular patterns (PAMPs) [12].

To the best of our knowledge, PFC nanoemulsions have not yet been used to target, visualize and monitor viral tumor infection and oncolytic virotherapy of tumors. Consequently, in this proof-of-principle study ^19^F MRI was applied to analyze tumor-bearing mice treated with the oncolytic Vaccinia virus (VACV) GLV-1h68. This oncolytic virus strain was previously described as a powerful agent to treat various types of cancer [29]–[31]. The presented results suggest ^19^F MRI as a non-invasive methodology to document the tumor-associated host immune response as well as the extent of intratumoral viral replication.

## Materials and Methods

### Ethics Statement

All animal experiments were carried out in accordance with protocols approved by the Institutional Animal Care and Use Committee (IACUC) of Explora Biolabs (San Diego, CA, USA, protocol number EB08-003) or the government of Unterfranken (Würzburg, Germany, protocol number AZ 55.2-2531.01-17/08). Both the Institutional Animal Care and Use Committee of Explora Biolabs and the government of Unterfranken specifically approved this study.

### Cell Lines

The human 1936-MEL melanoma cell line was originally isolated from late-stage metastases of patient 888 [32] and kindly provided to us by F. M. Marincola (National Institutes of Health, Bethesda, MD, USA). 1936-MEL cells were cultured in RPMI-1640 supplemented with 10% fetal bovine serum (FBS), 100 Units/ml penicillin, and 100 µg/ml streptomycin (PAA Laboratories, Cölbe, Germany). Human A549 lung carcinoma cells (ATCC No. CCL-185) were cultured in RPMI-1640 containing 10% FBS and 100 Units/ml penicillin, and 100 µg/ml streptomycin. GI-101A human ductual breast adenocarcinoma cells [33], [34] were kindly provided by A. Aller (Rumbaugh-Goodwin Institute for Cancer Research, Inc., FL, USA) and cultured in RPMI-1640 supplemented with 5 ng/ml β-estradiol and 5 ng/ml progesterone (Sigma Aldrich, Taufkirchen, Germany), 1 mM sodium pyruvate, 10 mM HEPES, 20% FBS, 100 Units/ml penicillin, and 100 µg/ml streptomycin. All cells were maintained at 37°C and 5% CO_2_.

### Virus

The construction of the attenuated Vaccinia virus strain GLV-1h68 was previously described by Zhang et al. [31]. Briefly, three expression cassettes (encoding for *Renilla* luciferase-GFP fusion protein, β-galactosidase and β-glucuronidase) were recombined into the *F14.5L*, *J2R* and *A56R* loci, respectively, of the LIVP strain virus genome. Viruses were propagated in CV-1 cells and purified through sucrose gradients.

### Tumor Inoculation, Administration of the Virus, Application of Perfluorocarbon (PFC)

Six-week-old female athymic nude *Foxn1^nu^* mice were obtained from Harlan Laboratories (Boxmeer, Netherlands or Indianapolis, IN, USA). 1936-MEL (7×10^5^/100 µl PBS), A549 (5×10^6^/100 µl PBS), and GI-101A tumor cells (5×10^6^/100 µl PBS) were subcutaneously (s.c.) injected into the abdominal right flank. At different time points after injection, the tumor volume was calculated as length×width^2^×0.52. For all experiments, tumors were grown up to 100–300 mm^3^ in size before viral administration. A single viral dose of 1×10^7^ plaque forming units (p.f.u.) in 100 µl PBS was injected intravenously (i.v.) via the tail vein.

The emulsified ^19^F perfluorocarbon (PFC) solution (VS-1000H, Celsense, Inc., Pittsburgh, PA, USA) with a mean particle size of approximately 145 nm [18] was directly i.v. injected as 20% v/v emulsion (100 µl) into tumor-bearing mice. The injection time points of each individual experiment are indicated in the corresponding figure legend.

### *In vivo* MRI

*In vivo* ^1^H and ^19^F MRI measurements were performed on a 7 Tesla Bruker Biospec System (Bruker BioSpin GmbH, Reinstetten, Germany) at room temperature using a 40 mm diameter home-built double resonant ^1^H/^19^F birdcage. For *in vivo* imaging the animals received inhalation anesthesia (1–2% isoflurane) during the measurement. Animals were placed together with a reference tube containing a mixture of the above described PFC emulsion and H_2_O in a home-built measurement container according to safety regulations.

In all experiments ^1^H MRI was performed prior to ^19^F imaging to enable correlation of the ^19^F signal distribution into the anatomical context. Thus, 3D ^1^H turbo-spin-echo (TSE) and 3D ^1^H multi-spin-echo (MSE) experiments were performed. For *in vivo* ^19^F imaging 3D TSE sequences were performed after the ^1^H experiments.

The parameters for the different ^1^H *in vivo* experiments are listed in table 1. No averaging was applied when ^1^H data was acquired.

**Table 1:** Parameters for the different *in vivo* ^1^H MRI experiments.

Model	Fig	Sequence	TE [ms]	TR [ms]	TF	NE	MTX	FOV [mm]
1936-MEL	1 B,C,D	TSE	30	1000	10	1	100×150×125	20×30×25
1936-MEL	3 B/4 A	TSE	30	1000	10	1	100×150×125	20×30×25
A549	5 A,B	MSE	4.4*	250	1	28	64×96×80	20×30×25

Acronyms: Fig = figure, TE = echo time, TR = repetition time, TF = turbo factor of TSE experiments, NE = number of echoes for MSE experiments, MTX = imaging matrix, FOV = field-of-view.

* In case of the MSE experiments TE labels the echo time of the first echo and the inter echo time.

The parameters for the different ^19^F *in vivo* animal experiments are presented in table 2. For each animal the total *in vivo* MRI protocol time was <2h.

**Table 2:** Parameters for the different *in vivo* ^19^F MRI experiments.

Model	Fig	Sequence	TE [ms]	TR [ms]	TF	NA	MTX	FOV [mm]
1936-MEL	1 B,C,D	TSE	4	1000	48	60	40×48×32	20×30×25
1936-MEL	3 B/4 A	TSE	4	1000	48	60	40×48×32	25×30×20
A549	5 A,B	TSE	4	1000	48	75	40×48×32	25×30×20

Acronyms (in addition to table 1): NA = number of averages.

### *Ex vivo* MRI

After completion of the *in vivo* experiments the 1936-MEL tumors were excised and snap-frozen in liquid N_2_ and stored at −80°C. For *ex vivo* ^19^F MRI measurements followed by histology the tumors were fixed in 4% paraformaldehyde/PBS pH 7.4 for 16 h at 4°C and rinsed in PBS followed by embedding and dehydration in 10% Sucrose/5% w/v low-melting point agarose/PBS.

In a first set of experiments, focusing on the 1936-MEL tumor model, all infected tumors (n = 3) were placed together in a single 15 ml tube (Greiner Bio-One GmbH, Frickenhausen, Germany) embedded in 10% Sucrose/5% w/v low-melting point agarose/PBS. The corresponding control tumors (n = 3) were placed in an additional 15 ml tube. Both tubes were placed together in the measurement coil to enable measurement of all tumor specimens in only one ^1^H and one ^19^F scan. The same embedding procedure was used for all other *ex vivo* measurements, however, for those experiments tumor specimen were placed in individual tubes (50 ml tubes: 1936-MEL; 15 ml tubes: GI-101-A) together with a reference tube containing PFC emulsion and H_2_O. Thus, for these tumor samples *ex vivo* MRI was performed individually. The same hardware as for *in vivo* MRI was used for the *ex vivo* measurement of 1936-MEL tumor specimen.

*Ex vivo* ^1^H and ^19^F MRI measurements of the GI-101A samples were performed on a 11.7 Tesla Bruker AMX System (Bruker BioSpin GmbH, Reinstetten, Germany) at room temperature using a 17 mm diameter home-built double-tunable ^1^H/^19^F birdcage.

Similar to *in vivo* measurements, ^1^H 3D TSE experiments were used to obtain anatomical background information. *Ex vivo* ^1^H experiments were followed by^ 19^F 3D TSE imaging. The chosen ^1^H and ^19^F *ex vivo* imaging parameters are presented table 3 and 4. No averaging was applied for *ex vivo* ^1^H MRI experiments.

**Table 3:** Parameters for the different *ex vivo* ^1^H MRI experiments.

Model	Fig	Sequence	TE [ms]	TR [ms]	TF	MTX	FOV [mm]
1936-MEL	1 E,F/1S A,B	TSE	81	1000	20	300×200×100	60×40×20
1936-MEL	2 B/4 C	TSE	35.5	1000	10	200×150×125	40×30×25
GI-101A	5 C,D	TSE	34	500	8	192×128×128	30×20×20

Acronyms (see table 1 and 2).

**Table 4:** Parameters for the *ex vivo* ^19^F MRI experiments.

Model	Fig	Sequence	TE [ms]	TR [ms]	TF	NA	MTX	FOV [mm]
1936-MEL	1 E,F/1S A,B	TSE	5	1000	64	450	96×64×32	60×40×20
1936-MEL	2 B/4 C	TSE	4.3	1000	48	90	64×48×40	40×30×25
GI-101A	5 C,D	TSE	6.2	1000	64	60	96×64×64	30×20×20

Acronyms (see table 1 and 2).

### Post Processing of MRI Datasets

Post processing was performed using home-written routines in MATLAB (The MathWorks Inc., Natick, MA, USA) if not mentioned otherwise.

#### Figure preparation

For anatomical correlation of the *in vivo* A549 ^19^F data, a summed image of all echo images was calculated for each ^1^H MSE experiment. Prior to summation, the echo images were pixel-wise weighted with the calculated T_2_ time assuming a mono-exponential decay (S(t) = S_0_ exp(−t/T_2_); S(t) = signal; t = time).

All ^19^F datasets were zerofilled to the matrix sizes of the respective ^1^H datasets. The SNR of the zerofilled ^19^F data were calculated following reference [35] for low SNR data. For better visualization the maximum of the shown 2D images of the 3D ^19^F datasets were scaled to a specific maximal SNR which is indicated in the respective figure legends. For ^1^H/^19^F overlay images a SNR threshold of 4.5 was chosen for the ^19^F data. Additionally, remaining, isolated ^19^F signal pixels were removed.

Regarding the 3D overlay reconstruction, ^19^F data were zerofilled as described above. The 3D ^1^H and the zerofilled ^19^F data were scaled to their respective maxima. The zerofilled 3D ^19^F data were set to a threshold as described above. Afterwards a 3D overlay dataset was generated. The overlay dataset was additionally masked by only selecting the animal area. The animal mask was manually generated with the help of the ^1^H 3D data. After the described pre-preparation of the 3D overlay data in MATLAB, the data were transferred to MeVisLab (MeVis Medical Solutions AG, Bremen, Germany) where the 3D surface reconstruction was performed. For the visual correlation of the *ex vivo* MRI data and histology results only tissue slices from the tumor middle were compared.

#### Quantitative analysis of the ^19^F MRI data

For quantitative analysis of the *in vivo* MRI time course in 1936-MEL tumor-bearing mice, ratios of the total ^19^F SNR of the tumor and the tumor volume (^19^FTVR) were generated. Since from each group only n = 2 animals were measured at 9 dpi, this parameter was only calculated for 7 and 11 dpi (n = 4 animals per group). The tumor regions were manually selected with the help of the fully resolved ^1^H images. SNR maps of the zerofilled ^19^F data were generated as described above. Only ^19^F signal pixels within the predefined regions-of-interest (ROI) having an SNR≥4.5 were regarded and single ^19^F signal pixels were removed. The total ^19^F SNR was evaluated by summation of all pixels remaining after the pre-preparation/−selection procedure. The ^19^F SNR data were additionally normed to the mean SNR of the corresponding reference. For each animal the corresponding ^19^FTVRs of 7 and 11 dpi were individually regarded. Thus, for each pair (day 7 and 11), the ^19^FTVRs were normed to the maximum value and the slope between both values was calculated assuming a linear relationship.

Similar as for *in vivo* MRI of 1936-MEL tumor-bearing animals, ratios of the total ^19^F SNR of the tumor and the tumor weight (^19^FTWR) were generated for *in vivo* MRI of A549 tumors and *ex vivo* MRI of 1936-MEL/GI-101-A tumor-bearing animals. Thus, SNR maps of the original ^19^F data were generated for quantitative analysis as described above. Overlays were generated with the help of the original ^19^F data (^19^F SNR≥4.5) and downscaled ^1^H data. This was done to select ROIs which excluded ^19^F signal from other regions (e.g. lymph nodes).

Additionally, for VACV infected animals having 1936-MEL tumors a correlation of the *ex vivo* ^19^F signal area with the GFP positive area was performed. Thus, for each tumor sample, the middle slice of the tumors was chosen using the original ^19^F data. Furthermore, ROIs of the tumor region were manually selected with the help of downscaled ^1^H data. In a next step, the number of signal pixels in the ROIs having a SNR≥4.5 was calculated. Single ^19^F signal pixels were removed. In a final step, the percentage of the ^19^F signal containing volume in regard to the tumor volume of the selected slice was calculated.

### Immunohistochemistry

Following *ex vivo* ^19^F MRI measurements histology was performed. Briefly, the paraformaldehyde-fixed and in 10% Sucrose/5% w/v low-melting point agarose/PBS embedded tumors were further dehydrated in 30% sucrose/PBS for 12 h and finally embedded in Tissue-Tek® O.C.T. (Sakura Finetek Europe B.V., Alphen aan den Rijn, Netherlands). Tumor samples were sectioned (15 µm) with the cryostat 2800 Frigocut (Leica Microsystems GmbH, Wetzlar, Germany). Antibody-labeling was performed following fixation in ice-cold acetone. The primary antibody was incubated for 1 h. After washing with PBS, sections were labeled for 30 min with the secondary antibody and finally mounted in Mowiol 4–88.

### Fluorescence Microscopy

The fluorescence-labelled preparations were examined using the MZ16 FA Stereo-Fluorescence microscope (Leica) equipped with the digital DC500 CCD camera and the Leica IM1000 4.0 software (1300×1030 pixel RGB-color images) as well as the Leica TCS SP2 AOBS confocal laser microscope equipped with an argon, helium-neon and UV laser and the LCS 2.16 software (1024×1024 pixel RGB-color images). Digital images were processed with Photoshop 7.0 (Adobe Systems, Mountain View, CA, USA) and merged to yield overlay images.

### Fluorescence Intensity and Immune Cell Density Measurements

The fluorescence intensity of the CD68-labelling or the percentage of the CD68-positive tumor area in 15-µm-thick cryostat sections of control and GLV-1h68-colonized tumors was measured on digital images (x10 objective or x40 objective, x1 ocular) of specimens stained for CD68 immuno-reactivity. On the fluorescence microscope, the background fluorescence was set to a barely detectable level by adjusting the gain of the CCD camera before all the images were captured with identical settings. The fluorescence intensity of the CD68-labelling was determined in RGB images and represented the mean brightness of all pixels (intensity range 20–255) and was measured using ImageJ software (http://rsbweb.nih.gov/ij). The whole area of the tumor cross-section was determined by manually drawing ROIs using ImageJ.

The immune cell density was determined either in microscopic images of whole tumor cross-sections (x10 objective, x1 ocular) by quantification of the positive labelled area using ImageJ software or by direct counting of CD68-positive immune cells in multiple areas of different tumor sections obtained with the confocal laser microscope (x20 objective, x10 ocular). On the fluorescence microscope, for each image the fluorescence signal was set to a clearly detectable level by individually adjusting the gain of the CCD camera before the images were captured. All RGB-images were equally adjusted and converted into 8-bit gray scale images using Photoshop 7.0. Positive pixels (intensity range 0–255) of the CD68-labelling were measured using ImageJ software. The immune cell density was shown as positive area/tumor section for whole tumor sections or as number of positive cells/field of view and presented as mean values with standard deviations.

### Antibodies

Immune cells were labeled either using rat anti-mouse CD68 (monocytes, tissue macrophages, dendritic cells; Abcam, Cambridge, UK), rat anti-mouse CD11b (myeloid cells, NK, T^act^, B cell subset; eBioscience, San Diego, CA, USA), rat anti-mouse MHCII antibody (B cells, dendritic cells, monocytes, macrophages; eBioscience) and rat-anti Ly-6G antibody (mainly neutrophils, myeloid cells; eBioscience).

The Cy3-conjugated secondary antibodies (donkey) were obtained from Jackson ImmunoResearch (West Grove, PA, USA). Hoechst 33342 (Sigma Aldrich) was used to label nuclei in tissue sections.

### Statistics

A two-tailed Students *t* test was used for statistical analysis of different quantitative parameters described above. Pearsotns rank correlation was used to determine the correlation coefficient r. *p* values of <0.05 were considered statistically significant.

## Results

### PFC Accumulates in 1936-MEL Melanomas during Oncolytic Virotherapy

**Figure 1:**
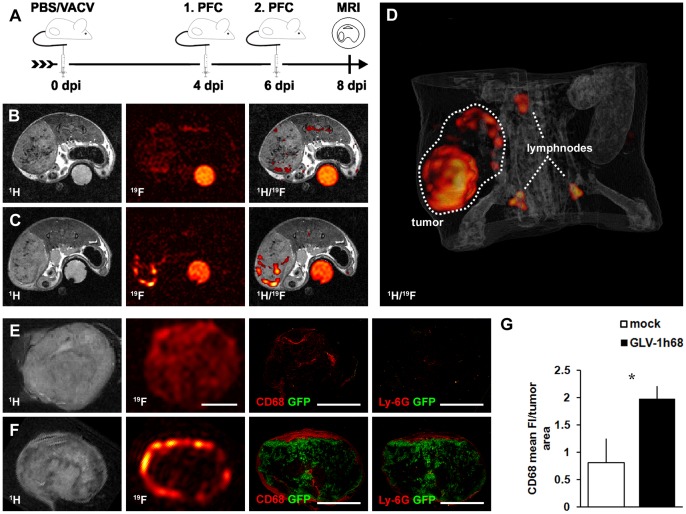
Visualization of viral tumor colonization by ^19^F MRI-based imaging of inflammation. (**A**) timeline - 1936-MEL melanoma-bearing athymic nude mice were i.v. injected with either 1×10^7^ pfu of GLV-1h68 or PBS as control. Emulsified PFC was i.v. administered at day 4 and 6 pi followed by ^19^F MRI at 8 dpi. (**B, C**) representative ^1^H, ^19^F, and overlayed ^1^H/^19^F *in vivo* images of a mock-infected tumor-bearing mouse (**B**) showing a low ^19^F signal throughout the tumor tissue with few signal hot spots and a corresponding GLV-1h68-infected tumor-bearing mouse (**C**) revealing intense ^19^F signal accumulation in the tumor rim 8 dpi. (**D**) 3D ^1^H/^19^F overlay reconstruction of the abdomen of a VACV-infected mouse clearly showed the partial/hollow sphere-like accumulation of PFC at the tumor margin and the adjacent lymph nodes. (**E, F**) representative *ex vivo* ^1^H, ^19^F images as well as corresponding histologically prepared tumor sections (CD68, Ly-6G) demonstrating the distribution of the CD68^+^-macrophage/Ly-6G^+^-neutrophil population and the ^19^F signal of a mock-infected tumor (**E**) and a GLV-1h68-infected tumor (**F**); GLV-1h68 infection corresponds to the GFP-expressing tumor area. (**G**) GLV-1h68-colonized tumors showed increased CD68-fluorescence intensity (FI) compared to mock-infected control tumors as determined in histologically prepared tumor sections (n = 3; p = 0.026). Shown are the mean values +/− standard deviations. The ^19^F signal intensity in B and C was scaled to SNR = 30 and in E and F to SNR = 75. The asterisk indicates a significant difference between experimental groups (*p<0.05; Students *t* test). All images are representative examples. Scale bars represent 5 mm.

To test the applicability of ^19^F MRI to monitor virus-induced inflammation during oncolytic virotherapy, we used 1936-MEL melanoma-bearing mice and administered VACV (GLV-1h68) i.v. followed by i.v. injection of emulsified PFC at day 4 and day 6 pi and MR imaging at 8 dpi (Fig. 1A). The tumor area was localized by ^1^H images followed by acquisition of anatomically matching ^19^F images in mock-infected control (Fig. 1B, n = 2) as well as VACV-treated animals (Fig. 1C, n = 2). The corresponding ^1^H/^19^F overlay *in vivo* images revealed intense hot spots of the ^19^F signal located at the tumor periphery in VACV-colonized animals compared to only few ^19^F-positive hot spots in the mock-infected control animals. The 3D ^1^H/^19^F overlay reconstruction of the abdomen of a VACV-infected mouse clearly showed an accumulation of PFC at the tumor margin (partial/hollow sphere) enabling tumor localization as well as localization of the adjacent lymph nodes (Fig. 1D). *Ex vivo* ^1^H/^19^F MRI imaging of excised tumors followed by CD68- and Ly-6G-immunohistology revealed a similar spatial distribution pattern of the ^19^F signal and CD68-positive monocytes/macrophages in both groups of mice (Fig. 1E, F, n = 3). However, in VACV-colonized tumors the macrophage-containing hot spots (^19^F^+^/CD68^+^) accumulate around the viral infected GFP-positive areas in the tumor periphery, whereas in control animals the CD68-positive macrophages and the corresponding ^19^F signal were generally distributed with lower signal intensity throughout the tumor tissue. Moreover, there were also Ly-6G-positive neutrophils located around the viral infected GFP-positive areas forming the inner part of the invading immune cell front. Analysis of the CD68-fluorescence signal intensity of whole tumor cross-sections by histology revealed a significantly enhanced fluorescence intensity in VACV-infected compared to mock-infected tumors (Fig. 1G; n = 3, p = 0.026).

The detailed histological analysis further showed that the ^19^F-positive/CD68-positive areas were also positive for CD11b and MHCII which confirm the affiliation to monocyte/macrophage populations (Figure S1).

Altogether, the results demonstrate that non-invasive ^19^F MRI can specifically visualize VACV-mediated spatial changes in the myeloid cell populations of tumors which may either due to recruitment or redistribution of tumoral myeloid cells.

### PFC and CD68^+^-macrophage Accumulation in 1936-MEL Melanomas during the Early Time Course of Infection (0–8 dpi)

**Figure 2:**
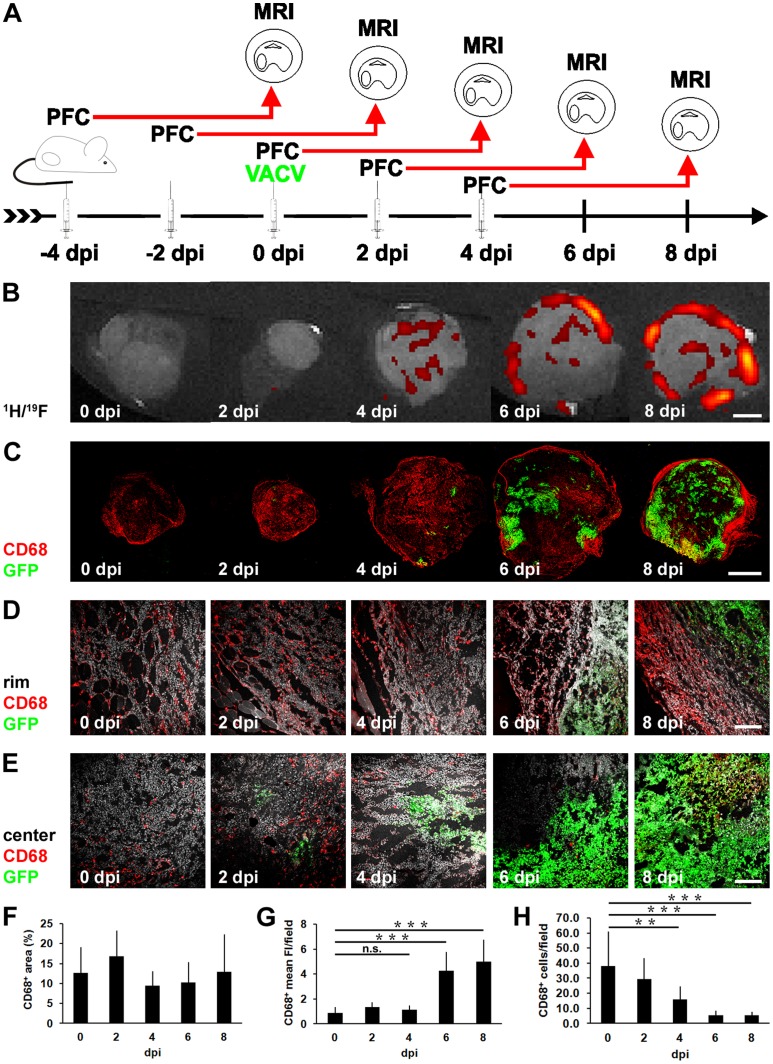
PFC and CD68^+^-macrophage accumulation during early infection time course (**0–8 dpi**). (**A**) timeline - 1936-MEL melanoma-bearing athymic nude mice were i.v. injected with emulsified PFC in each case 4 days before tumors were harvested. 1×10^7^ pfu of GLV-1h68 was i.v. administered at day 0 and tumors were harvested for *ex vivo* ^19^F MRI at 0, 2, 4, 6 and 8 dpi (2 tumors/time point). (**B**) representative ^1^H/^19^F images of 1936-MEL tumors at 0, 2, 4, 6, and 8 dpi analyzed by *ex vivo* ^19^F MRI revealed first detectable ^19^F signal at day 4 pi further increasing at day 6 and 8 pi; 6 and 8 dpi-infected tumors showed enhanced accumulation of the ^19^F signal at the tumor rim. (**C, F**) corresponding histologically prepared tumor sections labelled with anti-CD68 antibody to visualize the tumoral macrophage population at day 0, 2, 4, 6, and 8 dpi revealed no significant difference in the total CD68^+^ tumor area (%) at the different time points (8 tumor sections/time point). (**D, G**) confocal-laser microscopic images of the corresponding tumor sections taken from the tumor periphery (rim) showed a similar density of CD68^+^-macrophages/CD68 fluorescence intensity (FI) in 0–4 dpi tumors and a significant increase at 6 and 8 dpi (12 tumor areas/time point). (**E, H**) corresponding confocal-laser microscopic images of tumor sections taken from the intratumoral tissue (center) showed a significant decrease in the density of CD68^+^-macrophages during the time course of infection (12 tumor areas/time point). Shown are the mean values +/− standard deviations. The signal intensity of all presented ^19^F images was scaled to SNR = 30. Asterisks indicate a significant difference between experimental groups (n.s. = not significant, **p<0.01, ***p<0.001; Students *t* test). All images are representative examples. Scale bars represent 2 mm (B, C) and 300 µm (D, E).

To identify the timing of PFC accumulation in 1936-MEL melanomas after VACV-treatment, we performed a time course study of PFC accumulation during early infection stages (0, 2, 4, 6, and 8 dpi). Each group was injected with one dose of PFC 4 days before tumors were harvested and *ex vivo* MRI was performed (Fig. 2A). Immediately before (0 dpi) and 2 days pi no ^19^F signal was detectable by MRI (Fig. 2B). However, 4 days pi the first ^19^F-positive patches were detectable in the VACV-treated tumors which further increased at day 6 and 8 pi (Fig. 2B). Interestingly, in contrast to the 4 dpi tumors the distribution pattern of the ^19^F hot spots of the 6 and 8 dpi tumors differed and ^19^F hot spots were mostly located in close proximity to the tumor rim. Therefore, for robust detection of viral-induced ^19^F accumulation we suggest 2–4 dpi as the optimal injection time point for PFC in 1936-MEL tumor-bearing mice followed by ^19^F MR imaging 6–8 days post VACV injection.

To confirm the MRI results, we performed immuno-histological analysis of the CD68^+^-macrophage population in tumor sections at all time points. CD68-positive cells were detectable in all investigated tumors with no significant difference of the total CD68-positive tumor area during the infection time course (Fig. 2C, F). Interestingly, the distribution pattern of the CD68-positive cells was notably different in 0–4 dpi and 6–8 dpi tumors changing from an intra- and peritumoral scattered pattern to a strong accumulation of macrophages at the tumor margin. Detailed analysis of the intratumoral and the peripheral tumor areas revealed a significant increase in the CD68^+^-macrophage population in the tumor margin concomitant with increasing viral spreading (Fig. 2D, G) and a significant loss of the intratumoral CD68^+^-macrophage population (Fig. 2E, H).

### Long-term Imaging of PFC Accumulation – a Spatio-temporal Analysis

**Figure 3:**
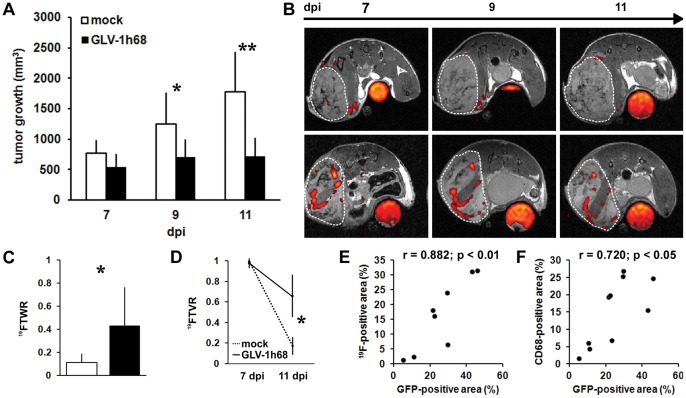
Long term PFC accumulation and correlation to viral tumor load. The mice were either mock-infected or treated with 1×10^7^ pfu GLV-1h68 (i.v.) and i.v. injected with emulsified PFC at day 4 pi. (**A**) 1936-MEL tumor growth was monitored by measuring the tumor volume of 7–8 mice in each group revealing a continuous tumor growth in the mock-infected group and a stable disease in the GLV-1h68-treated group. Shown are the mean values +/− standard deviations. (**B**) representative ^1^H/^19^F images of 1936-MEL tumor-bearing mice either mock-infected (upper row) or treated with GLV-1h68 (lower row) and i.v. injected with emulsified PFC at day 4 pi. *In vivo* ^19^F MRI was performed at day 7, (9), and 11 pi of n = 4 (n = 2) mice of each group. (**C**) quantitative evaluation of the ^19^FTWRs of *ex vivo* ^19^F MRI measurements of mock-infected and GLV-1h68-treated 1936-MEL tumors at 11 dpi revealed a significant difference between both groups (n = 7/8; p = 0.031). (**D**) quantitative evaluation of the ^19^FTVR decrease (7 to 11 dpi) of mock-infected and GLV-1h68-treated 1936-MEL tumors during the time course study measured by *in vivo* ^19^F MRI revealed a significant difference between both groups (n = 4; p = 0.038). (**E, F**) significant positive correlation of either the amount of the ^19^F-positive area determined in 625 µm-thick MRI sections (**E**) or the CD68-positive area determined in 15 µm-thick histological sections (**F**) with the extent of viral infection as determined by the GFP-expressing area in corresponding 15 µm-thick tumor sections (Pearsońs correlation coefficient PFC/GFP: r = 0.882; p = 0.004; CD68/GFP: r = 0.720; p = 0.044). Shown are the mean values +/− standard deviations. The signal intensity of all presented ^19^F images was scaled to SNR = 30. Asterisks indicate a significant difference between experimental groups (*p<0.05, **p<0.01; Students *t* test). All images are representative examples.

To analyze the stability and/or the spatial distribution of the ^19^F signal pattern over time, we injected PFC only once at day 4 pi in mock- and VACV-treated groups of 1936-MEL tumor-bearing mice and performed ^19^F MRI at day 7, 9, and 11 dpi. During these time points tumor growth arrests in VACV-treated animals in contrast to the continuously increasing tumor volume of the mock-infected group (Fig. 3A). In each group the ^19^F signal distribution pattern was similar during the imaging period (Fig. 3B). However, the ^19^FTWR (total ^19^F SNR of the tumor/tumor weight) was significant higher in VACV-treated at 11 dpi compared to mock-treated tumor-bearing animals as determined by *ex vivo* ^19^F MRI (Fig. 3C; n = 7–8, p = 0.031). Moreover, quantification of the ^19^F signal over time revealed a tendential ^19^FTVR (total ^19^F SNR of the tumor/tumor volume) signal decrease during the time course in both tested groups, in which the mock-infected group showed a significant higher decrease than the VACV-treated group (Fig. 3D; n = 4, p = 0.038).

Importantly, both the ^19^F-positive area (Fig. 3E; r = 0.882, p = 0.004) as well as the CD68-positive area (Fig. 3F; r = 0.720, p = 0.044) in tumor sections of VACV-treated animals positively correlated with the degree of VACV-infection as determined by the GFP-positive tumor area enabling indirect intratumoral virus detection.

### Non-macrophage-related PFC Accumulation in Large, Mock-infected Control Tumors

**Figure 4:**
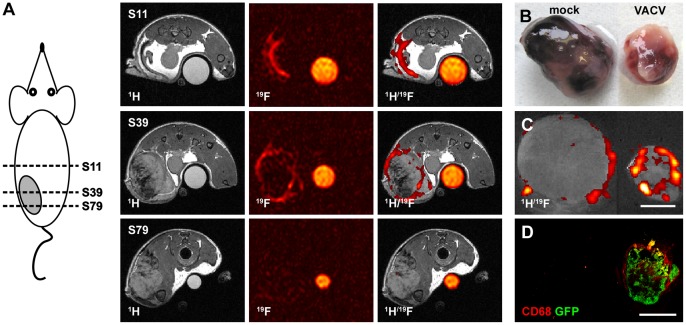
PFC also accumulates in peritumoral hematomas of mock-infected animals bearing large 1936-MEL tumors independently of CD68^+^-macrophages. (**A**) mock-infected 1936-MEL tumor-bearing mouse i.v. injected with emulsified PFC at 4 dpi. *In vivo* ^19^F MRI 7 dpi revealed an accumulation of the ^19^F signal in axial slices of the abdomen far-out from the tumor region (S11), in some tumorous sections (S39), whereas other tumor-containing sections did not reveal ^19^F accumulation (S79). (**B**) morphological analysis of PFC-accumulating mock-infected animals revealed large hematomas in the peritumoral areas compared to GLV-1h68-treated tumors. (**C, D**) *ex vivo* ^1^H/^19^F images of mock-infected and GLV-1h68-infected tumors revealed a similar distribution pattern of the ^19^F signal (**C**) and the CD68^+^-macrophage population (**D**) only for the GLV-1h68-treated tumors. The signal intensity of all presented ^19^F images was scaled to SNR = 30. All images are representative examples. Scale bars represent 5 mm.

During our study, we noticed that some large, mock-infected tumors can incorporate substantial PFC amounts after intravenous PFC application. This could interfere with the reliable detection of virus-mediated immune cell recruitment. However, this group of mainly large tumors showed a different distribution pattern of the ^19^F signal compared to the previously shown scattered intratumoral ^19^F pattern of mock-infected control tumors as shown in Fig. 1E. The analysis of different ^1^H/^19^F overlay axial slices of the abdomen revealed that the ^19^F signal accumulation also appeared in non-tumorous areas and was mostly located outside of the malignant tissue in abdominal cross-sections (Fig. 4A). Interestingly, the investigation of the phenotype of these large, mock-infected tumor-bearing animals revealed that these mice developed peritumoral hematoma and even bloody tumors in comparison to VACV-treated tumor-bearing mice (Fig. 4B). Direct comparison of the ^1^H/^19^F signal pattern of large, mock-infected tumors and VACV-infected tumors by *ex vivo* MRI showed that the ^19^F signal accumulated in the outermost part of the tumor rim in the mock-infected animals, whereas ^19^F hot spots accumulated deeper into the tumor tissue in VACV-treated animals (Fig. 4C). Histological analysis revealed a similar pattern of CD68^+^-macrophage populations and ^19^F hot spots only for the VACV-treated tumors and no matching was found for mock-infected tumors (Fig. 4D).

### ^19^F-MRI-based Imaging of VACV-induced Recruitment of Macrophages in Different Tumor Models

**Figure 5:**
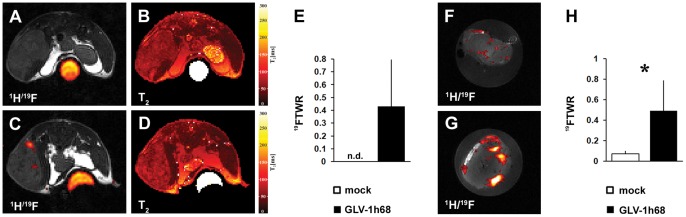
GLV-1h68-induced accumulation of intratumoral PFC is also detectable in lung carcinomas and breast adenocarcinomas. (**A–D**) ^1^H/^19^F overlay and ^1^H T_2_ images of mock-infected (**A, B**) and GLV-1h68-infected (**C, D**) A549 lung carcinoma-bearing mice were analyzed 7 dpi by *in vivo* ^19^F MRI (PFC, 4 dpi). (**E**) quantitative evaluation of the ^19^FTWR of *in vivo* ^19^F MRI measurements of mock-infected and GLV-1h68-treated A549 tumors at 7 dpi revealed a detectable ^19^FTWR only in VACV-treated tumors (n = 4). (**F–H**) mock-infected (**F**) and GLV-1h68-infected (**G**) GI-101A breast adenocarcinoma-bearing mice were analyzed 10 dpi by *ex vivo* ^19^F MRI (PFC injection, 7 dpi). (**H**) quantitative evaluation of the ^19^FTWR of *ex vivo* ^19^F MRI measurements of mock-infected and GLV-1h68-treated GI-101A tumors at 10 dpi revealed a significant difference between both groups (n = 4–5; p = 0.033). Shown are the mean values +/− standard deviations. The signal intensity of all presented ^19^F images was scaled to SNR = 30. Asterisk indicate a significant difference between experimental groups (n.d. = not detectable; *p<0.05; Students *t* test). All images are representative examples.

Since different tumor types as well as individual tumors of the same origin can greatly vary in their content of phagocytic macrophages, the polarization of the macrophage status and the immune-related response to therapy [36], [37], we decided to test our concept in different tumor models. Thus, we chose the A549 lung carcinoma model bearing few macrophages as well as the GI-101A breast adenocarcinoma model harboring a substantial population of tumor-associated macrophages before treatment (unpublished results). Despite these immunological differences, in both tumor types a significant increase in the ^19^F signal due to VACV-treatment was detectable in comparison to the mock-infected animal group (Fig. 5, Figure S2).

In addition we wanted to emphasize, that the use of ^19^F PFC as cellular contrast agent should not prevent the utilization of quantitative ^1^H parameters. Exemplarily, ^1^H T_2_ maps were acquired for the A549 lung carcinoma model (Fig. 5B, D) indicating the possible integration of other quantitative ^1^H MRI parameters to facilitate tumor characterization [38].

In summary, the ^19^F-MRI-based imaging of tumoral VACV-colonization is applicable in different tumor models and possesses high potential for monitoring oncolytic therapy. However, the great diversity of the microenvironment of different tumor models and even individual tumors implies for future work a specific and thus personalized imaging modality applying ^19^F MRI before and after starting the oncolytic treatment.

## Discussion

We have shown that the VACV-induced infiltration of myeloid cells into different tumor models is reliably detectable by ^19^F MRI. Therefore, we suggest that ^19^F MRI may be used in the future as a novel tool to quantitatively and non-invasively monitor the innate immune response in tumors following oncolytic virotherapy. Thus, this imaging technique can be readily investigated as a surrogate measure to monitor viral tumor colonization and also therapeutic response. To the best of our knowledge, this is the first report of utilizing PFC nanoparticles in combination with ^19^F MRI for detection of myeloid cell infiltration into tumors undergoing oncolytic virotherapy. Other investigators have reported the use of PFC nanoparticles to monitor inflammatory conditions in different other pathological situations [13]–[18]. Recently, Kleijn [39] used a Gadolinium (Gd)-based agent targeting the inflammatory enzyme myeloperoxidase (MPO) in a similar approach to detect oncolytic virus-associated tumor inflammation by MRI. However, ^19^F MRI has several advantages compared to contrast agent based ^1^H MRI including no background in tissues [20], the possibility of direct quantification of the marker amount [24] and the unaltered ^1^H signal making the assessment of other quantitative ^1^H parameters more feasible [18].

In the present study, ^19^F MRI as well as the histological examination of mock-infected tumors revealed a diffuse distribution of both the ^19^F signal and the CD68^+^-macrophages throughout the tumor. In this respect, the so called enhanced permeability and retention (EPR) effect, which is a microenvironmental characteristic of solid tumors leading to the passive and unspecific accumulation of a variety of macromolecules and nanoparticles, may enhance the accumulation of PFC in large mock-infected control tumors [40]. However, the direct comparison to VACV-treated tumors clearly showed that the viral tumor colonization significantly altered the ^19^F signal distribution as well as the signal intensity. In VACV-treated tumors the ^19^F-positive hot spots, which showed a similar distribution pattern as the CD68^+^-macrophage population, encapsulated the viral infection focus and formed a “partial/hollow sphere” localized to the tumor rim. In this regard, 3D-reconstructions of the abdominal region of VACV-treated tumor-bearing mice enable a straightforward localization of the tumorous regions. Since GLV-1h68 preferentially colonizes metastases [41] this imaging modality may be useful to detect metastases. In accordance with previous findings [13], [17], we further observed a strong ^19^F signal in the adjacent lymph nodes of tumors in 1936-MEL-bearing animals also indicating the potential of this imaging modality for the localization of sentinel lymph nodes. In future studies, we will especially analyze ^19^F-positive lymph nodes and clarify whether these lymph nodes are already metastasized or sites of an ongoing inflammatory immune cell activation.

Recent studies combining blood density gradient centrifugation and ^1^H/^19^F MRI showed that i.v. applied PFCs are predominantly taken up within the blood stream by monocytes but to a minor degree also by B-cells and neutrophils [13], [15]. After uptake, especially PFC-labelled neutrophils and macrophages migrate to sites of ongoing inflammation, where monocytes/macrophages are the predominant PFC-labelled cell fraction [15], [19]. In ^19^F-positive hot spots of VACV-treated tumors both monocyte-derived macrophages (CD68, CD11b, MHCII) as well as neutrophils (Ly-6G) were detectable, however, clearly spatially separated in different areas of the invading immune cell front. Neutrophils were localized in the inner part of the immune cell immigrants, whereas macrophages were located in the surrounding outer area. Since neutrophils are the first actors at the infection site followed by monocytes/macrophages [42] the here observed accumulation pattern of both immune cell populations implies that both populations are recruited in a timely different fashion to the VACV-infected tumor rather than re-distributed within the tumor tissue.

The time course study from 0 to 8 dpi based on ^19^F MRI and histological analysis of VACV-treated tumors revealed discrepancies between the spatio-temporal ^19^F accumulation and the CD68^+^-macrophage population during the course of infection. The ^19^F signal was first detectable at 4 dpi further increasing in the tumor rim at day 6 and 8 pi, whereas the CD68^+^-macrophage population was at all investigated time points detectable, however, either distributed throughout the whole tumor (0–4 dpi) or mostly located with a high density at the tumor rim (6–8 dpi). Further, the microscopic analysis revealed that the intratumoral macrophage population significantly decreases whereas the peritumoral population increases during the course of infection and with increasing viral spreading. These results indicate that the resident TAM population may be directly eliminated from the tumor tissue by viral infection and simultaneously a second VACV infection-induced population of ^19^F-positive macrophages was recruited from the circulation to the tumor encapsulating the infection focus. The lack of the ^19^F accumulation early after infection may be responsible either to the lower phagocytic activity of the resident macrophage population [37] or to the reduced vessel permeability in these tumors avoiding significant intratumoral accumulation of PFC after injection. Since the presence of TAMs in several cancer types correlates strongly with a poor outcome [43] studies were already performed to develop a clinically applicable, non-invasive diagnostic assay for visualization of TAMs in tumors based on MRI and clinically applicable iron oxide nanoparticles [44]. In the present study, however, the results indicate that, rather than labelling the intratumoral TAMs, PFC nanoparticles may label mainly the immune cells in the circulation which immigrate into the tumor after viral colonization.

The observed decrease of the ^19^FTVRs during the time course study could be mainly explained by the continuous tumor growth in the mock-infected group. However, in VACV-treated animals the average tumor volume remained stable during the imaging period. Therefore, the decrease in the ^19^FTVRs in VACV-treated mice may be either due to an emigration of PFC-labelled macrophages to e.g. draining lymph nodes, which could also explain the observed ^19^F signal in the sentinel lymph nodes (Fig.1 D) or to the loss of freely accumulated PFC via efferent lymphatics. For the immuno-therapeutic effect of oncolytic virotherapy, emigration of phagocytic cells from the tumor to adjacent lymph nodes may offer a way to activate an adaptive anti-tumoral immune response via tumor antigen presentation to B- and T cells and should be further investigated in future studies.

The examination of the ^19^F-positive area and the extent of the viral spreading determined by the GFP-expression revealed a significant positive correlation for both parameters indicating that ^19^F MRI during oncolytic virotherapy enables indirect monitoring of viral replication. This would be beneficial for non-invasive monitoring of intratumoral viral replication in pre-clinical and in future clinical studies, since ^19^F MRI provides a whole organ visualization circumventing the problems associated with small sample biopsies such as false-negative or -positive results of viral replication. For future studies using ^19^F MRI for monitoring of oncolytic virotherapy, we suggest a specific personalized imaging modality applying ^19^F MRI before and after starting the oncolytic treatment to avoid misinterpretation of unspecific PFC accumulation as we have shown in tumor-associated hematoma.

For correlation purposes only tissue slices from the tumor middle and the same orientation were always chosen for histological sections and ex-vivo ^19^F images. However, due to the different slice thickness and possible alignment errors of the histological section and the MRI data, the correlation remains limited. In the future, as has been done with ^1^H MRI [45], sophisticated ^19^F MRI coils should be developed to improve the correlation of ^19^F MRI and histology.

Since we could show that ^19^F MRI enables the detection of viral tumor colonization and immune cell recruitment to tumors in different tumor types with different immunological context, we assume that the here described imaging modality may also be useful in the future for clinical applications. Thorne previously discussed that it is also likely that systemic measurements of the level and type of immune response induced by the viral treatment may ultimately be used either as an early prognostic indicator of therapeutic response, or may help elucidate the immune properties of the tumor being treated, and so assist in the design of subsequent immunotherapeutic treatments [46]. In the same manner, we propose that ^19^F MRI may have the potential to be used for therapeutic monitoring and as prognostic indicator for the therapeutic outcome.

## Supporting Information

Figure S1 **Co-localization of the ^19^F signal with monocytes/macrophages and neutrophils.** Representative *ex vivo* ^1^H/^19^F overlays as well as corresponding histologically prepared tumor sections demonstrating a similar distribution pattern of the CD68^+^- (monocytes/macrophages), MHCII^+^- (antigen-presenting cells such as dendritic cells (DCs), macrophages and B cells), CD11b^+^- (myeloid cells), Ly-6G^+^-population (neutrophils) and the ^19^F signal of a mock-infected tumor **(A)** and a GLV-1h68-infected tumor **(B)**. The ^19^F signal intensity was scaled to SNR = 75. All images are representative examples. Scale bars represent 5 mm.
(TIF)

Figure S2 **GLV-1h68-induced accumulation of intratumoral PFC and CD68 histology of breast adenocarcinomas. (A–D)** ^1^H/^19^F overlay images (left row) and CD68 histology (right row) of mock-infected **(A, B)** and GLV-1h68-infected **(C, D)** GI-101A breast adenocarcinoma-bearing mice analyzed 10 dpi by *ex vivo* ^19^F MRI (PFC injection, 7dpi). GFP corresponds to GLV-1h68 infection of the tumor tissue. The signal intensity of all presented ^19^F images was scaled to SNR = 30. All images are representative examples.
(TIFF)
