# Supplementary figures and images for: Imaging of Intratumoral Inflammation during Oncolytic Virotherapy of Tumors by 19F-Magnetic Resonance Imaging (MRI)
Source: PLoS One. 2013 Feb 18;8(2):e56317. doi: 10.1371/journal.pone.0056317 (PMC3575337; doi:10.1371/journal.pone.0056317)

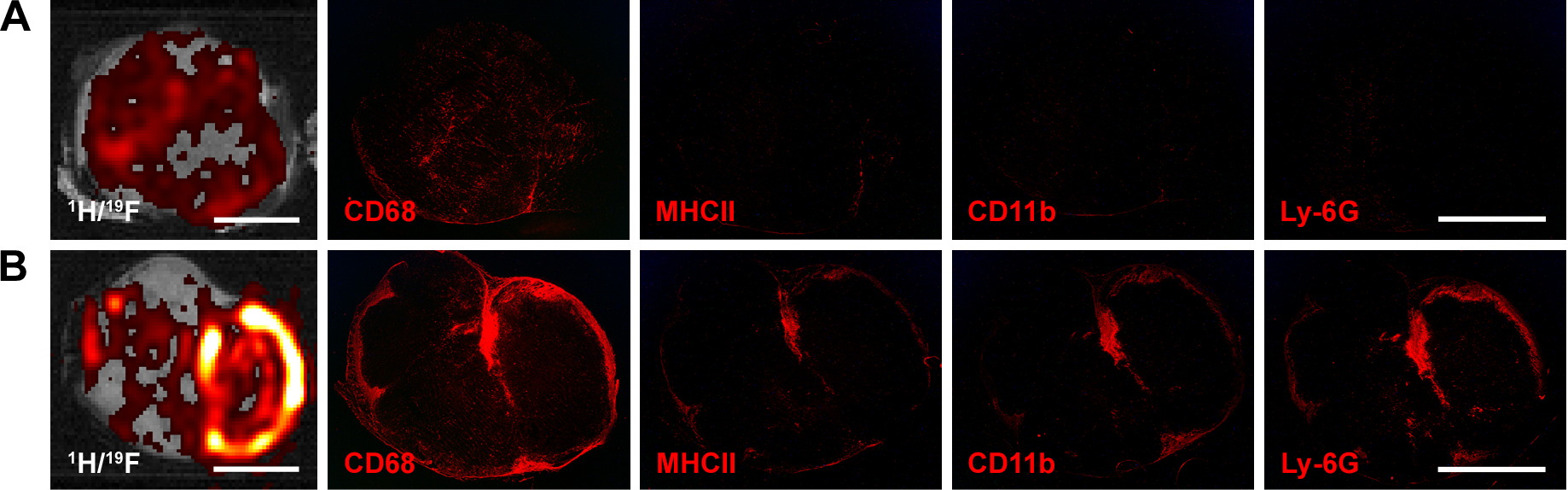

Supplement: Figure S1 — Co-localization of the 19F signal with monocytes/macrophages and neutrophils. Representative ex vivo 1H/19F overlays as well as corresponding histologically prepared tumor sections demonstrating a similar distribution pattern of the CD68+- (monocytes/macrophages), MHCII+- (antigen-presenting cells such as dendritic cells (DCs), macrophages and B cells), CD11b+- (myeloid cells), Ly-6G+-population (neutrophils) and the 19F signal of a mock-infected tumor (A) and a GLV-1h68-infected tumor (B). The 19F signal intensity was scaled to SNR = 75. All images are representative examples. Scale bars represent 5 mm. (TIF) [file pone.0056317.s001.tif]

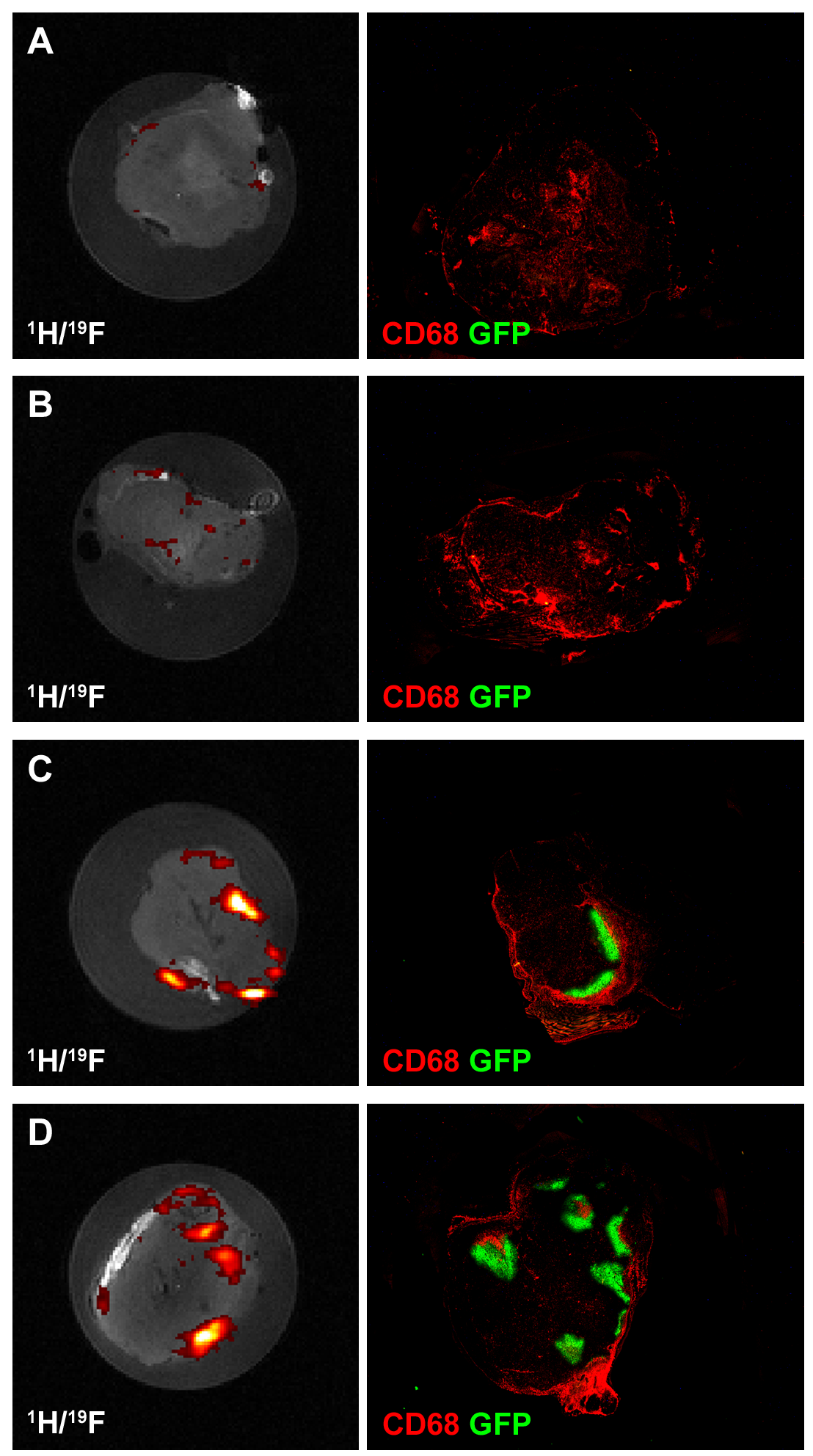

Supplement: Figure S2 — GLV-1h68-induced accumulation of intratumoral PFC and CD68 histology of breast adenocarcinomas. (A–D) 1H/19F overlay images (left row) and CD68 histology (right row) of mock-infected (A, B) and GLV-1h68-infected (C, D) GI-101A breast adenocarcinoma-bearing mice analyzed 10 dpi by ex vivo 19F MRI (PFC injection, 7dpi). GFP corresponds to GLV-1h68 infection of the tumor tissue. The signal intensity of all presented 19F images was scaled to SNR = 30. All images are representative examples. (TIFF) [file pone.0056317.s002.tiff]
